# Development and analytical validation of a novel bioavailable 25-hydroxyvitamin D assay

**DOI:** 10.1371/journal.pone.0254158

**Published:** 2021-07-09

**Authors:** Anders H. Berg, Mahtab Tavasoli, Agnes S. Lo, Sherri-Ann M. Burnett-Bowie, Ishir Bhan, S. Ananth Karumanchi, Sahir Kalim, Dongsheng Zhang, Sophia Zhao, Ravi I. Thadhani

**Affiliations:** 1 Department of Pathology, Cedars Sinai Medical Center, Los Angeles, California, United States of America; 2 Department of Pathology, Beth Israel Deaconess Medical Center and Harvard Medical School, Boston, Massachusetts, United States of America; 3 Department of Medicine and Biomedical Sciences, Cedars-Sinai Medical Center, Los Angeles, California, United States of America; 4 Division of Nephrology and Center for Vascular Biology Research, Department of Medicine, Beth Israel Deaconess Medical Center and Harvard Medical School, Boston, Massachusetts, United States of America; 5 Endocrine Division, Department of Medicine, Massachusetts General Hospital, Boston, Massachusetts, United States of America; 6 Division of Nephrology, Department of Medicine, Massachusetts General Hospital, Boston, Massachusetts, United States of America; University Sains Malaysia, MALAYSIA

## Abstract

**Background:**

Bioavailable 25-hydroxyvitamin D (25OHD) may be a better indicator of vitamin D sufficiency than total 25OHD. This report describes a novel assay for measuring serum bioavailable 25OHD.

**Methods:**

We developed an assay for 25OHD % bioavailability based on competitive binding of 25OHD tracer between vitamin D-binding protein (DBP)-coated affinity chromatography beads and serum DBP. Bioavailable 25OHD, total 25OHD, albumin, and DBP protein concentrations were measured in 89 samples from hospitalized patients and 42 healthy controls to determine how the DBP binding assay responds to differences in concentrations of DBP and compares to calculated bioavailable 25OHD values.

**Results:**

DBP binding assay showed a linear relationship between DBP-bound 25OHD tracer recovered from bead supernatant and DBP calibrator concentrations (y = 0.0017x +0.731, R^2^ = 0.9961, p<0.001). Inversion of this relationship allowed interpolation of DBP binding equivalents based upon 25OHD tracer recovered. The relationship between DBP binding equivalents and % bioavailability fits a non-linear curve, allowing calculation of % bioavailable 25OHD from DBP binding equivalents (y = 10.625x^-0.817^, R^2^ = 0.9961, p<0.001). In hospitalized patient samples, there were linear relationships between DBP protein concentrations and DBP binding equivalents (y = 0.7905x + 59.82, R^2^ = 0.8597, p<0.001), between measured vs. calculated % bioavailability (y = 0.9528 + 0.0357, R^2^ = 0.7200, p<0.001), and between absolute concentrations of measured vs. calculated bioavailable 25OHD (y = 1.2403 + 0.1221, R^2^ = 0.8913, p<0.001).

**Conclusions:**

The DBP-binding assay for bioavailable 25OHD shows expected changes in 25OHD % bioavailability in response to changes in DBP concentrations and concordance with calculated bioavailable 25OHD concentrations.

## Introduction

The “free hormone hypothesis” claims that only hormone not bound by high affinity binding proteins are bioavailable to target tissues [1–4]. One implication of this is that the measurement of the total concentrations of protein-bound ligands may not be the best indicator of whether concentrations are physiologically adequate [1, 3, 5]. The majority of circulating 25OHD is tightly bound by Vitamin D binding protein (DBP) [6, 7]. The fraction of 25OHD not bound by DBP has been called “bioavailable” which includes both free 25OHD and 25OHD loosely bound by albumin and other plasma proteins, and it has been proposed that only this unbound fraction is bioavailable for conversion to active 1,25-dihydroxyvitamin D [1, 2]. This role for DBP may also imply that the concentration of circulating DBP is thus a limiting factor for how much 25OHD can be carried in circulation and may influence the half-life and circulating concentrations of total 25OHD. Although the high affinity of DBP for 25OHD allows it to act as a carrier, it also likely limits the bioavailability of 25OHD to target tissues. Mice and at least one patient genetically deficient for DBP have been shown to have profoundly decreased plasma concentrations of 25OHD which were not corrected by vitamin D supplementation, however this patient and mice showed no clinical signs of Vitamin D deficiency, suggesting that DBP deficiency increased bioavailable 25OHD concentrations enough to support calcium homeostasis despite very low total 25OHD concentrations [8–10]. Recently our group reported that serum total concentrations of 25OHD are strongly correlated with concentrations of DBP in critically ill patients [4]. In medical conditions where low concentrations of 25OHD are also associated with low concentrations of DBP, low DBP concentrations should increase the bioavailability of 25OHD, thus suggesting that these patients will have proportionately higher concentrations of bioavailable 25OHD and may not suffer from functional 25OHD insufficiency [4, 11].

Measurement of bioavailable 25OHD (which we define as the proportion of 25OHD not bound to DBP) may be a better indicator of 25OHD sufficiency. In previous studies by our group, we used methods for calculating concentrations of bioavailable 25OHD based upon measured concentrations of total 25OHD, DBP, and albumin and published estimates for their respective binding affinity constants. We reported that calculated bioavailable 25OHD concentrations were more strongly associated with bone mineral density than total 25OHD concentrations in healthy subjects, and that bioavailable 25OHD was more strongly associated with plasma calcium and parathyroid hormone concentrations than were total 25OHD concentrations in patients on hemodialysis [12, 13]. A number of subsequent studies by others have since corroborated the association between bioavailable 25OHD and indicators of calcium homeostasis as well as cardiovascular health [14–17].

It is important to note that the hypothesis that measurement of free and/or bioavailable 25OHD are better biomarkers for vitamin D adequacy than total 25OHD is still controversial, and the data are not always consistent [3]. One of the reasons for these mixed results may be analytical limitations of existing methods for measurement of bioavailable 25OHD. Previous investigations of bioavailable 25OHD revealed caveats to the accuracy of methods for estimating bioavailable 25OHD based upon measurement of total 25OHD and DBP and estimated affinity binding constants between 25OHD and DBP [18–20]. Calculated estimations of free and bioavailable 25OHD assume that the binding affinities between DBP, albumin, and 25OHD are accurate. There are three common protein variants present in the population, Gc1S, Gc1F, and Gc2. Early studies of the binding between DBP and 25OHD indicated that binding affinities differed significantly between the three variants, suggesting that calculation of bioavailable 25OHD required modification based on variant-specific binding affinities [21–24]. None of these studies observed comparable binding affinities amongst the individual variants, and therefore these studies provided consistent evidence that the different variants had different binding affinities, however the true binding affinities of each variant are not really known. Other caveats to calculating bioavailable 25OHD include the challenge of calculating bioavailable 25D in patients heterozygous for two different DBP variants with different binding affinities, and also problems accurately measuring DBP concentrations using immunoassays that may not detect DBP variants equally [25, 26]. Together these challenges suggest that accurately measuring bioavailable 25OHD may require an assay method which physically separates bioavailable and DBP-bound 25OHD fractions.

Traditionally, the most commonly used methods for separation and measurement of free hormone concentrations are equilibrium dialysis and centrifugal ultrafiltration [5, 27]. 25-hydroxyvitamin D is especially hydrophobic, and despite many attempts our lab has never been able to observe measurable amounts of 25OHD tracer passing through a porous cellulose membrane using either of these methods. To the best of our knowledge, equilibrium dialysis and centrifugal ultrafiltration have only been reported for measurement of free 1,25-dihydroxyvitamin D (which is less hydrophobic and has much lower affinity for DBP) [27–29]. Our experience suggests that 25OHD is unable to pass through commercially available porous membranes.

**Fig 1:**
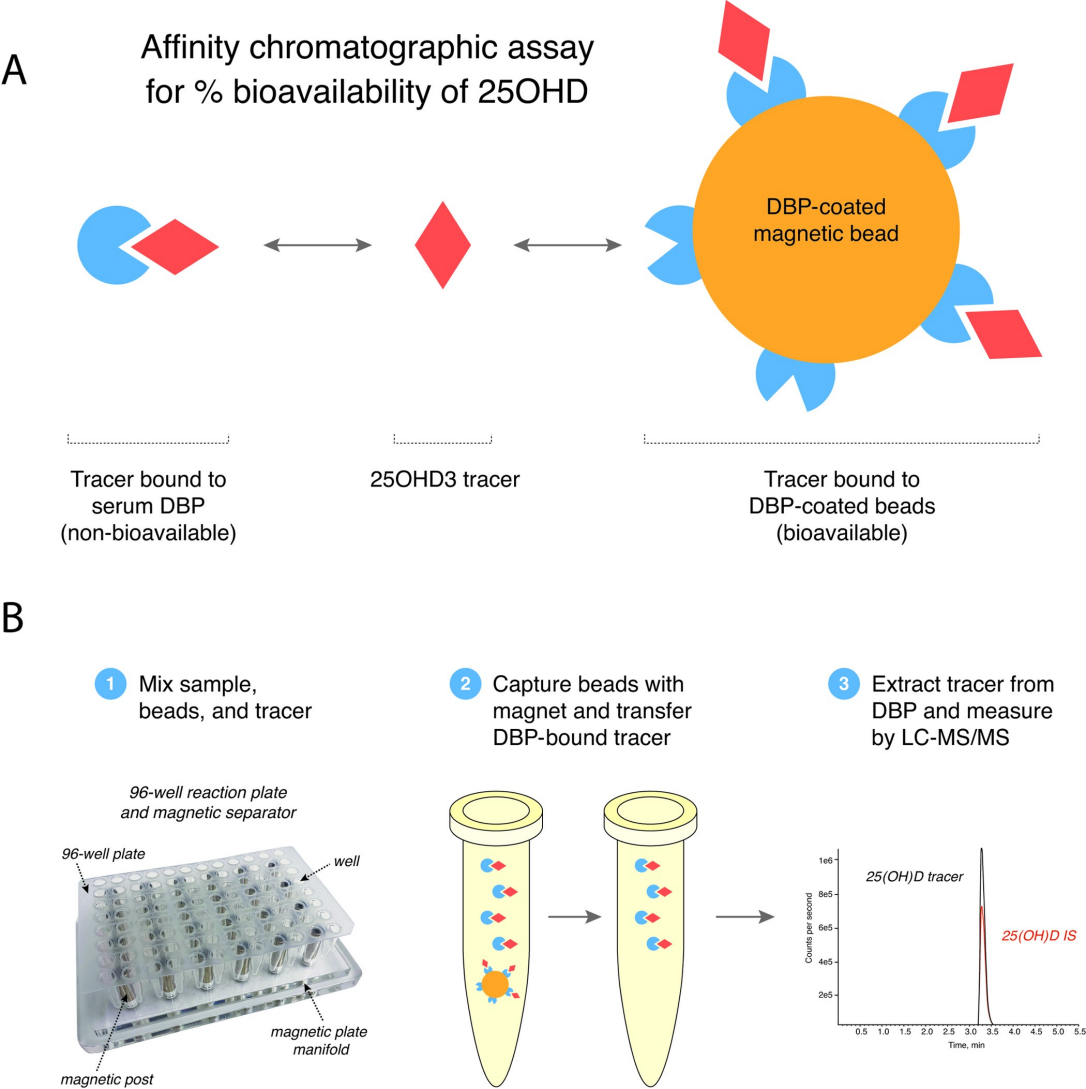
Assay for bioavailable 25OHD. **(A)** Graphic model of affinity chromatographic assay for % bioavailability of 25OHD. **(B)** Graphic illustration of methods for separation and measurement of DBP-bound 25OHD tracer from bioavailable 25OHD tracer by affinity chromatography on DBP-coated magnetic beads.

To bypass these analytical challenges, we utilized affinity chromatography as a method of physically separating bioavailable 25OHD from DBP-bound 25OHD. Serum is mixed with a fixed amount of deuterium-labeled 25OHD_3_ tracer and diluted with saline buffer at physiologic pH. This is mixed with magnetic beads coated with covalently attached purified DBP. The 25OHD tracer equilibrates between the patient’s serum DBP (i.e., DBP-bound and thus not bioavailable) and the immobilized DBP on the beads (Fig 1). The supernatant containing the DBP-bound non-bioavailable tracer is then separated from the beads, and the non-bioavailable 25OHD is extracted from the supernatant and measured by LC-MS/MS. Amounts of recovered tracer are then converted into “DBP-binding equivalents” from which we calculate the % bioavailability of 25OHD. Finally, the absolute concentrations of bioavailable 25OHD are calculated by multiplying % bioavailability by total 25OHD concentrations. The advantage of this method is that it relies on the binding equilibrium between DBP in the patient’s sample and purified DBP immobilized on the beads, and thus responds to changes in the concentration and binding affinity of patients’ circulating DBP. As a result, the assay accounts for differences in DBP binding affinities associated with DBP genetic variants [18].

## Materials and methods

### Study design and participants

This was a prospective observational study of hospitalized adults (age ≥18) recruited after admission to an intensive care unit. Acutely ill patients were specifically studied because of previous studies showing the association between acute illness and low DBP and 25OHD concentrations. Analysis of this study cohort has been previously published by our group; we specifically reported analysis of the association between serum DBP and serum total 25OHD concentrations from this cohort [4]; in this present report we instead focus on the relationships between serum DBP and bioavailable 25OHD measured using our newly developed DBP-binding assay [4]. Candidates who enrolled after providing written informed consent had samples collected daily for up to 7 days. These samples were originally collected for clinical purposes and only taken for research purposes after all clinical testing was completed. Thirty-six subjects were enrolled; the analyses were limited to the 25 subjects for which multiple daily samples were available. For comparison to healthy control subjects, DBP, 25OHD, and Bioavailable 25OHD concentrations were also measured in samples from the Metabolic Abnormalities in College Students (MACS) study, a study designed to evaluate the prevalence of metabolic abnormalities in university students [13]. Subjects were healthy 18- to 31-year-old male and female students from private universities in the Boston area. All subjects provided written informed consent. The study was approved by the MIT Committee on the Use of Humans as Experimental Subjects. Forty-two subjects had sufficient sample for inclusion in this analysis. None of the subjects received oral or intravenous vitamin D supplements during their hospital stay. Both hospitalized subject study and MACS study were conducted in compliance with the World Medical Association Declaration of Helsinki—Ethical Principles for Medical Research Involving Human Subjects, and approved by the Institutional Review Board of Massachusetts General Hospital and Massachusetts Institute of Technology, respectively.

### Data collection

Daily blood serum samples were collected on at least two and up to five days of each subject’s medical admission and analyzed for biochemical analytes as described below. Age, gender, BMI and medical diagnosis on admission were obtained from subjects’ electronic medical records at the time of enrollment. For the healthy control study, subjects were instructed to fast for 12 hours prior to admission to the MIT Clinical Research Center (CRC) and underwent a baseline evaluation including a blood sample collection and various physiologic measurements.

### Biochemical analyses

Blood samples were drawn into serum separator tubes without anticoagulant, centrifuged and separated serum stored at -80°C for future analysis. Concentrations of total 25OHD_3_ and 25OHD_2_ were measured from 100 μL of serum using LC-MS/MS using previously published methods, coefficients of variance for 25OHD_3_ and 25OHD_2_ assays were 1.1% and 1.3%, respectively [30]. Serum DBP was measured using LC-MS/MS; detailed methods for monitoring of quantification peptides are described in S1 File and S1 Table. The DBP LC-MS/MS assay was calibrated using purified DBP standard purchased from Sigma Aldrich (Gc-globulin, catalog no. G8764, Natick, MA); calibrators were made with phosphate buffered saline pH 7.4, human serum albumin 4.5 g/dL, and varying concentrations of DBP; DBP assay validation experiments are described in Supporting information and S1 Fig. Aliquots of pooled serum frozen at -80°C were used to monitor inter-assay quality control, the coefficient of variance of the assay was 6.2%. All LC-MS/MS assays were performed using an API 5000 triple quadrupole mass spectrometer (SCIEX, Framingham, MA) interfaced with a Shimadzu UPLC chromatography system with autosampler (Shimadzu USA, Columbia, MD). Intact serum PTH, serum total calcium, and serum albumin were measured in a CLIA-certified clinical laboratory using a Cobas 6000 automated analyzer (Roche Diagnostics, Indianapolis, IN, USA; inter-assay CVs: 2.5%, 3.8%, and 2.3% respectively). Immunoassay-based measurement of Free 25-dihydroxyvitamin D was measured by enzyme-linked immunoassay originally developed by Future Diagnostics (Wijchen, Netherlands); this assay has been used in a growing number of published studies [17, 31, 32]. Unless otherwise noted, all other chemicals were purchased from Sigma Aldrich (St. Louis, MO).

#### Measurement of bioavailable 25-hydroxyvitamin D

Measurement of bioavailable 25OHD was performed using a newly developed affinity chromatographic assay (Fig 1). The assay measures the proportion of 25OHD which is bound to patient’s DBP versus the fraction that is bioavailable 25OHD by mixing isotopic 25OHD_3_–[^2^H_3_] ligand with patient serum and with magnetic beads coated with covalently-linked purified DBP, all in a buffered saline to approximate physiologic binding conditions. The 25OHD tracer then binds competitively between the patients’ serum DBP and the bead bound DBP; the proportion of 25OHD tracer bound to DBP in solution represents the non-bioavailable fraction, and the fraction bound to DBP on the beads represents the bioavailable fraction (Fig 1A). After capturing the beads on a magnetic plate manifold (Fig 1B), the supernatant containing non-bioavailable 25OHD tracer is separated, mixed with 25OHD_3_–[^2^H_6_] isotopic internal standard, and the tracer is measured by LC-MS/MS. The assay is calibrated using standards containing 25OHD tracer with 4.5 g/dL human albumin and varying concentrations of purified DBP; in this way, amounts of non-bioavailable 25OHD tracer can be converted into DBP binding-equivalent units. The DBP-equivalent units are then converted into % bioavailable 25OHD values using the % bioavailable 25OHD values of the calibrators, which are calculated assuming an affinity binding constant of 7 x 10^8^ M^-1^ [24]. The absolute concentration of bioavailable 25OHD is then determined by taking the product of % bioavailable 25OHD and concentrations of total 25OHD. Additional method details and materials of this assay and its analytical validation are described in the Results and Supporting information.

### Statistical analysis

Baseline data were summarized using means and standard deviations. Percent (%) frequencies were computed for categorical variables. The tests of statistical significance for all analyses were set at a two-sided P<0.05.

## Results

### Responsiveness of assay to changes in DBP

**Fig 2:**
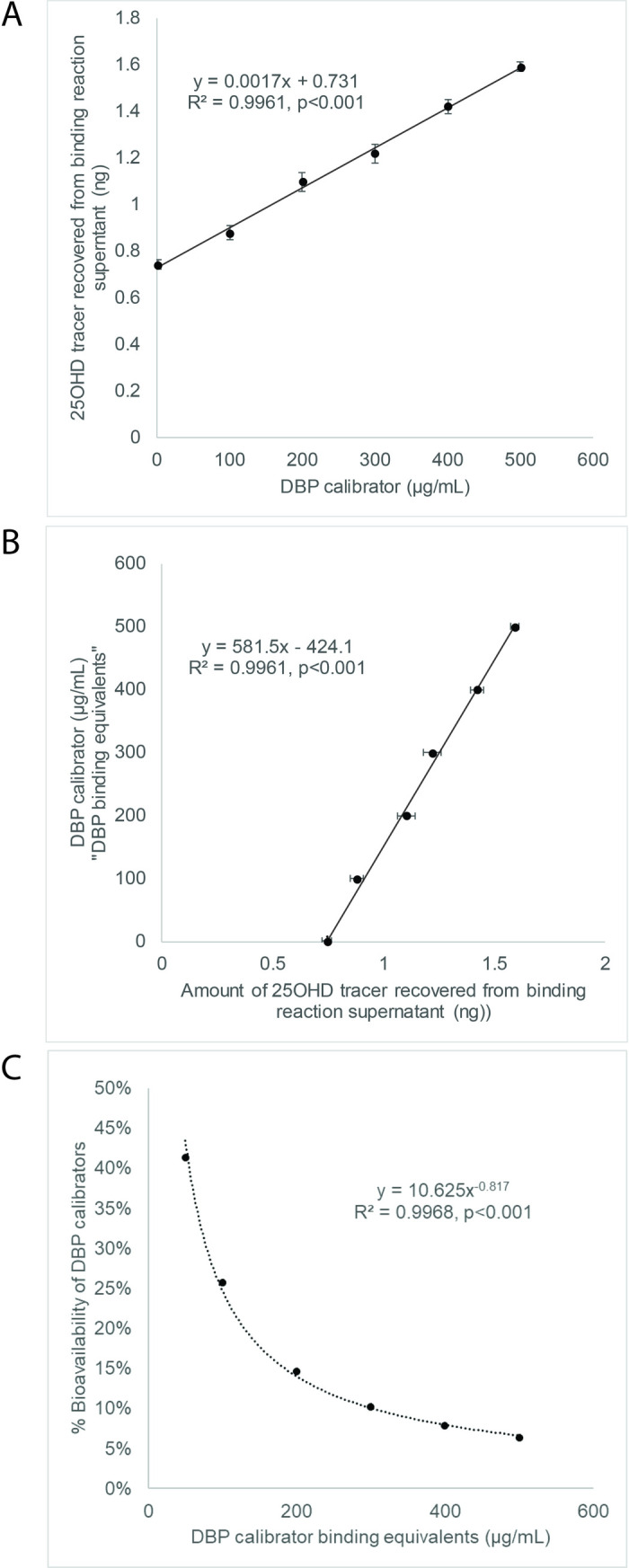
Assay for 25OHD—DBP binding equivalents. (A) Testing changes in 25OHD tracer recovery from binding assay supernatant in response to increasing concentrations of DBP calibrator. (B) Conversion of 25OHD tracer recovery from DBP calibrator samples into “DBP binding equivalent” concentrations. (C) Conversion of DBP calibrator binding equivalents into 25OHD % bioavailability.

After developing magnetic beads coated with covalently linked DBP and optimizing assay conditions for separation of serum bioavailable and non-bioavailable 25D (S2 and S3 Figs), we sought to test the assay’s responsiveness to difference in DBP concentrations. First, we measured the amounts of 25OHD tracer recovered from the binding reaction when increasing amounts of DBP were added (in triplicate). As shown Fig 2A, there is a linear increase in tracer recovered from bead supernatant when increasing concentrations of DBP were added to the reaction (y = 0.0017x +0.731, R^2^ = 0.9961, p<0.001 where y-axis values are amounts of 25OHD tracer recovered and x-axis are concentrations of DBP calibrator). When the x-y relationship was inverted, the relationship can be used as a calibration curve, converting recovered tracer into “DBP-binding equivalents” based upon the known concentrations of DBP calibrators (Fig 2B, y = 581.5x – 424.1, R^2^ = 0.9961, p<0.001 where y-axis values are DBP binding equivalent values and x-axis is amounts of 25OHD tracer recovered). To translate DBP-binding equivalents into the % bioavailability of 25OHD, we calculated the % bioavailability for our calibrators using previously published methods, assuming a generic DBP binding affinity constant of 7 x 10^8^ M^-1^, serum albumin concentration of 4.5 g/dL and albumin binding affinity of 6 x 10^3^ M^-1^ [13]. We found that the linear relationship between DBP binding equivalents and % bioavailability could be accurately modeled using a power function, and thus we could generate a calibration curve to convert DBP binding equivalents into % bioavailability values (Fig 2C, y = 10.625x^-0.817^, R^2^ = 0.9961, p<0.001 where y-axis values are 25OHD % bioavailability and x-axis values are DBP-binding equivalents). This calibrator curve was then used to convert the DBP binding equivalents measured in patient samples into their corresponding % bioavailability values. Note that during method development, assay measurements were performed in parallel at both room temperature and 37°C on purified DBP calibrator samples and a subset of patient serum samples; we observed no significant differences in DBP binding equivalents at either temperature (data not shown). As such, assays were thereafter performed at room temperature.

### Intra-assay precision

We measured the precision of our assay on DBP calibrators by measuring tracer recovery, DBP-binding equivalents, and corresponding % bioavailable 25OHD on our DBP standards at concentrations of 100 μg/mL and 500 μg/mL. The standard deviations and coefficients of variance for tracer recovery, DBP-binding equivalents, and % bioavailable 25OHD for our high concentration (100 μg/mL and 500 μg/mL) were measured and shown in S2 Table.

### Characteristics of binding assay in patient serum

After developing the assay for DBP-binding equivalents and % bioavailable 25OHD on purified DBP standards, we tested the characteristics of our assay on patient serum samples from a previously published cohort study [4]. Enrolled subjects included 25 patients who were admitted to a hospital intensive care unit for acute illness and consented to participate in the study; 23 of the subjects self-identified as white ethnicity and 2 subjects identifying as Asian descent. In order to compare results from critically ill patients to that of healthy controls, single samples from 42 additional healthy subjects enrolled in the previously published MACS study were analyzed for comparison [13]. Subject characteristics are shown in Table 1. ICU subjects provided between 2 and 5 samples drawn on separate days during their admission, resulting in a total of 89 samples for the ICU study. We chose to validate the assay on acutely ill patients because of prior studies showing that acute illness may be associated with abnormally low concentrations of 25OHD and DBP [33–37] and because of our recently published evidence suggesting that concentrations of 25OHD may be changing in response to changes in DBP in acutely ill patients [4]. In this previously published report, we observed that concentrations of serum 25OHD are strongly correlated with concentrations of DBP in acutely ill patients, and that the changes in DBP within individual patients coincided with parallel changes in 25OHD [4]. These findings would suggest that measurement of non-DBP bound bioavailable 25OHD (which should reflect concentrations of 25OHD and account for differences in DBP concentration and binding affinity) may better reflect vitamin D physiologic adequacy in acutely ill patients.

**Table 1:** Subject characteristics.

Characteristic	Mean value +/- SD	
	ICU subjects (n = 25)	Healthy controls (n = 42)
Age	61 (17)	23 (3)*
Female gender (%)	15 (60)	21 (50)*
BMI (kg/m^2^)	32.9 (10.2)	22.4 (3.0)*
Total 25OHD (ng/mL)	23.8 (18.1)	25.7 (11.1)
Total 25OHD (nmol/L)	59.5 (45.3)	64.3 (27.8)
Measured Bioavailable 25OHD (ng/mL)	2.2 (1.3)	2.7 (1.6)
Measured Bioavailable 25OHD (nmol/L)	5.5 (3.3)	6.8 (4.0)
DBP (μg/mL)	180 (86)	259 (159)*
DBP (μmol/L)	3.1 (1.5)	4.5 (2.7)*
Percent Bioavailable 25OHD (%)	11.6 (4.9)	11.5 (8.2)
Calcium (mg/dL)	8.5 (0.9)	9.2 (0.7)
Parathyroid hormone (pg/mL)	58 (44)	30 (8)*
Albumin (g/dL)	2.9 (0.4)	4.2 (0.4)*

*P<0.05.

**Fig 3:**
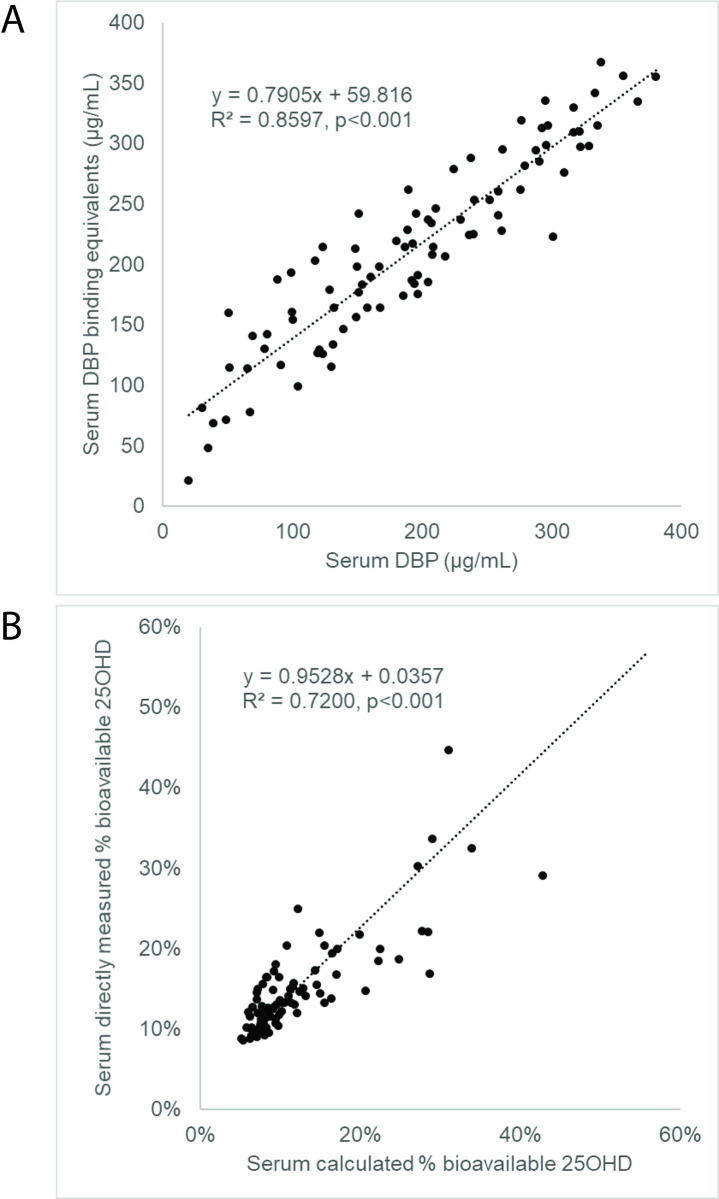
Linearity of relationships between serum DBP, DBP binding equivalents, and calculated % bioavailability of 25OHD. (A) Linear relationship between DBP binding equivalents measured by DBP-binding assay and serum DBP measured by LC-MS/MS. (B) Linear relationship between 25OHD % bioavailability measured by DBP-binding assay and calculated 25OHD % bioavailability.

**Fig 4:**
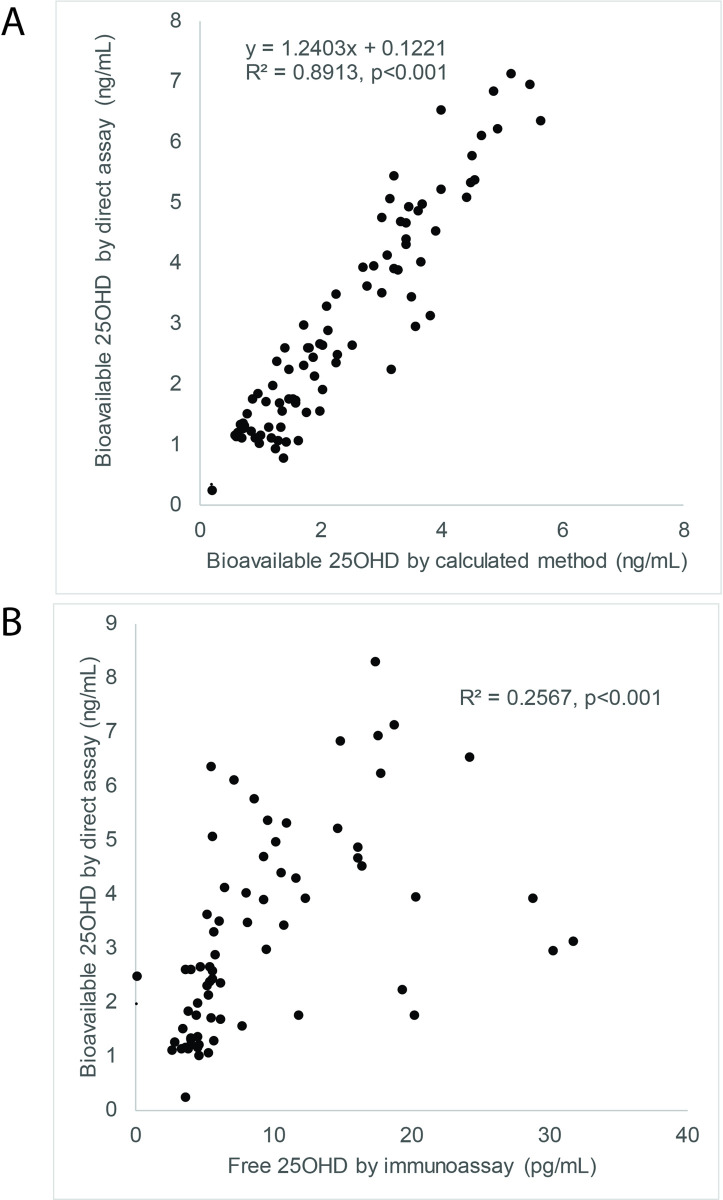
Linearity of relationships between calculated and measured concentrations of serum bioavailable 25OHD. (A) Linear relationships between bioavailable 25OHD measured by direct binding assay and calculated bioavailable 25OHD. (B) Linear relationships between bioavailable 25OHD measured by direct binding assay and free 25OHD measured by immunoassay.

To validate our assay for bioavailable 25OHD in patient samples, we tested the relationship between measured DBP protein concentrations and DBP-binding equivalents measured in patient serum samples. As shown in Fig 3A, there was a significant linear association and correlation between DBP protein concentrations and DBP-binding equivalents among ICU patients (y = 0.7905x + 59.82, R^2^ = 0.8597, p<0.001) and among healthy control subjects (y = 0.6186x + 47.60, R^2^ = 0.5363, p<0.001), validating the responsiveness of our binding assay to changes in DBP concentrations within a serum matrix. Furthermore, when we measured the % bioavailability of 25OHD using our DBP-binding assay and compared values to calculated % bioavailability (estimated based on concentrations of DBP and serum albumin) in ICU patients, we found a significant linear relationship and correlation between the two methods (y = 0.9528 + 0.0357, R^2^ = 0.7200, p<0.001) (Fig 3B). Although the methods correlated strongly, close inspection shows that methods differed significantly in some samples, suggesting that factors other than DBP concentration (e.g., DBP protein variant type) influenced the true bioavailability of 25OHD. When absolute concentrations of bioavailable 25OHD were determined based upon the product of total 25OHD concentrations and 25OHD % bioavailability, as shown in Fig 4A, we observed that measured bioavailable 25OHD assay produced values that were strongly correlated, but not identical to calculated bioavailable 25OHD (y = 1.2403 + 0.1221, R^2^ = 0.8913, p<0.001). Lastly, we also found that measured bioavailable 25OHD concentrations were also correlated to concentrations of “Free 25OHD” measured by a commercial immunoassay method (Fig 4B, R^2^ = 0.2567, p<0.001).

### Relationship between changes in concentrations of DBP and measured bioavailable 25OHD over time in acutely ill patients

**Fig 5:**
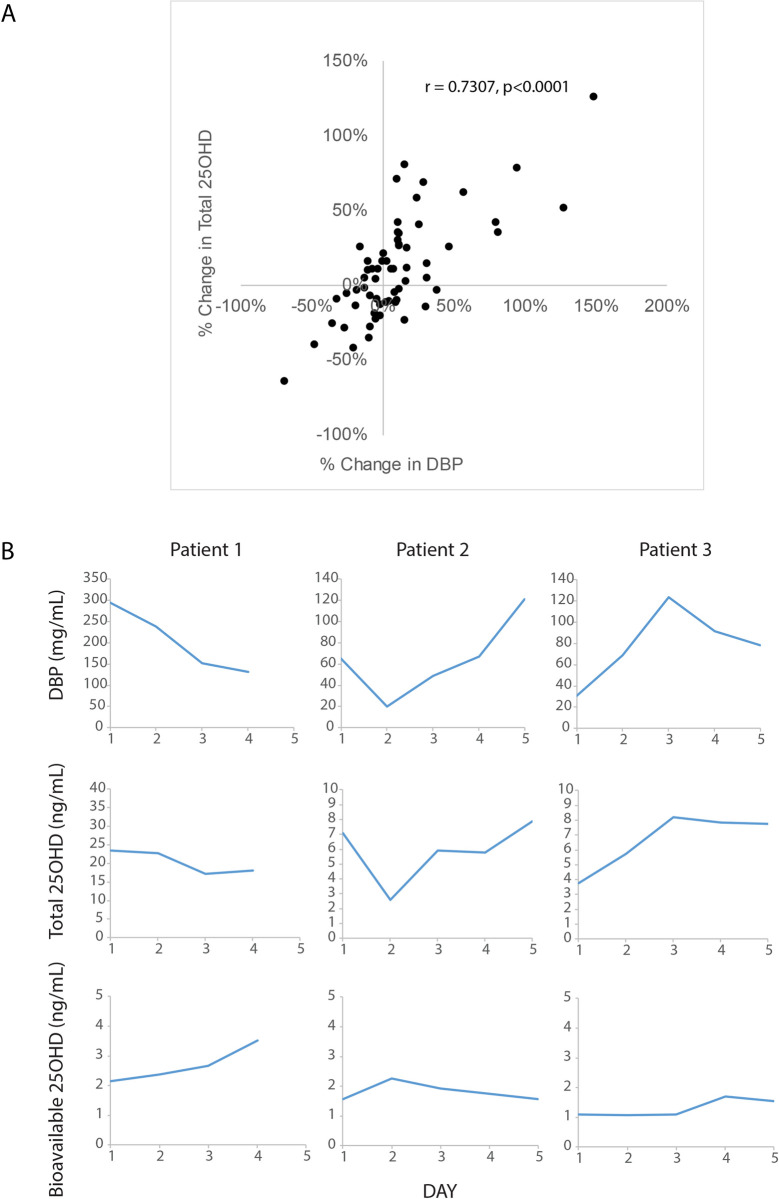
Relationship between changes in concentrations of DBP and measured bioavailable 25OHD over time in acutely ill patients. (A) Correlation between % changes over time in total 25OHD and % changes in DBP concentrations in ICU patients. (B) Time course graphs comparing changes in concentrations of DBP, Total 25OHD, and Bioavailable 25OHD during the weeklong study period in three representative patients.

Previously we have reported that concentrations of total 25OHD and DBP were significantly correlated in acutely ill patients, and often changed in parallel over time, whereas concentrations of free 25OHD measured by immunoassay remained comparatively more stable during hospitalization [4]. We investigated whether this same pattern was observed for measured bioavailable 25OHD in these sample patient samples. As shown in Fig 5A, the % changes in 25OHD over time amongst hospitalized patients were significantly correlated with % changes in serum DBP. To further illustrate this trend, subjects who experienced significant changes in serum DBP often experienced parallel changes in 25OHD, whereas the changes seen in bioavailable 25OHD were comparatively less, consistent with what had been observed previously for free 25OHD measured by immunoassay (Fig 5B) [4]. When we correlated the % change of 25OHD to the % change in DBP in serially collected samples (e.g. % change in 25OHD and DBP measured in samples from Patient 1 collected on day 1 compared to day 2, etc.), there was a significant correlation between % change in 25OHD and % change in DBP (r^2^ = +0.53, p<0.001). In contrast, there was no significant correlation between % change in DBP and % change in bioavailable 25OHD concentrations (r = 0.014, p = 0.27). Lastly, intra-individual variability in measured bioavailable 25OHD over time in these acutely ill patients was less pronounced compared to variation in total 25OHD (average coefficients of variance over time within individual patients of 12.3% vs. 18.6% for bioavailable vs. total 25OHD, respectively).

## Discussion

This report describes the development and analytical validation of a novel assay for bioavailable 25OHD which utilizes affinity chromatography to separate bioavailable 25OHD from DBP-bound 25OHD. Theoretical advantages of the assay are that it bypasses the caveats of porous membrane separation and uses DBP protein as a binding reagent which responds to effects of temperature, pH, and matrix in the same way as patients’ endogenous DBP. The relationship between concentrations of DBP in the sample and amounts of 25OHD tracer recovered from the binding assay are reproducibly linear, avoiding analytical challenges associated with non-linear assays, and allowing confident interpolation of results over the analytical measuring range. Analytical validation experiments on patient samples found that DBP binding equivalents correlated strongly with serum DBP concentrations, measured % bioavailability of 25OHD correlated with calculated % bioavailability, and absolute concentrations of measured bioavailable 25OHD correlated significantly with calculated concentrations of 25OHD. Importantly, differences in calculated vs. measured % bioavailable 25OHD in individual samples suggest that additional factors beyond DBP concentration (such as variability in DBP protein variant binding affinities or additional 25OHD binding factors) influence the true bioavailability of 25OHD.

We recently reported that concentrations of 25OHD parallel changes in concentrations of its primary plasma carrier protein in acutely ill patients [4]. This previous report as well as the data reported herein provide evidence suggesting a dependent relationship between circulating concentrations of 25OHD and DBP. In addition to binding 25OHD, DBP is also an actin binding protein that may be consumed during critical illness [33, 34]. In our study, the average concentrations of DBP measured using our LC-MS/MS assay was lower compared to the healthy control group (Table 1), and lower than the previously published average values in healthy patients, consistent with reports that DBP is decreased during critical illness [35]. The unusually low concentrations of total 25OHD seen in some of these patients are also similar to previous reports observing low 25OHD levels amongst acutely ill patients [35, 36], and in many of our patients, dramatic increases and decreases of 25OHD were seen even though no changes in vitamin D intake occurred during the study. We also observed that changes in DBP over time were often associated with parallel changes in total 25OHD within individual patients, that percent changes in DBP were strongly correlated with % changes in total 25OHD, and % changes in bioavailable 25OHD within subjects over time tended to be smaller than % changes in total 25OHD during hospitalization. Together these findings imply that acute changes in DBP may influence circulating concentrations of 25OHD in acutely ill patients, independent of vitamin D intake or biosynthesis, and acute changes in bioavailable 25OHD during acute illness may be comparatively smaller than the dramatic changes in total 25OHD that may be seen.

It was notable that comparison of ICU patients to healthy controls found that although average serum DBP concentrations were significantly lower in ICU patients compared to healthy controls (Table 1), the average % bioavailability of 25D measured using the DBP-binding equivalents assay did not differ significantly between groups. This may reflect the fact that ICU patients had not only lower DBP concentrations, but also significantly lower serum albumin; according to previously published models of hormone bioavailability [2], albumin-bound hormones are considered to be bioavailable, and thus when both DBP and albumin concentrations are low, there may be no net effect on % bioavailability.

The medical relevance of these findings may have important significance for testing and interpretation of 25OHD levels in acutely ill patients. Similar to evaluation of thyroid function in ICU patients, measured concentrations of 25OHD may be difficult to interpret in the ICU setting. Importantly, the majority of clinical trials testing the benefits of vitamin D supplementation in acutely ill patients with hypovitaminosis have not shown consistent benefits [37, 38]. Although this report constitutes an intriguing analytical validation of our assay for bioavailable 25OHD and its responsiveness to serum DBP concentrations, future studies in larger cohorts with well annotated clinical endpoints are required to determine whether bioavailable 25OHD is a better indicator of vitamin D adequacy and calcium homeostasis in acutely ill patients. It will be especially important to study whether assays for measured bioavailable 25OHD more accurately reflect Vitamin D sufficiency in patients known to have abnormally high or low DBP levels, for example in patients who are pregnant or have liver cirrhosis [10, 39, 40]. It is also important to note that these findings did not test the significance of bioavailable 25OHD concentrations amongst healthy patients who have generally stable and non-limiting concentrations of circulating DBP [41]; future studies are needed to determine whether bioavailable or total 25OHD concentrations best reflect vitamin D adequacy amongst genetically heterogenous populations.

## Conclusions

The DBP-binding assay for bioavailable 25OHD shows expected changes in 25OHD % bioavailability in response to changes in DBP concentrations and concordance with calculated bioavailable 25OHD concentrations in calibrators made with purified DBP as well as in real patient serum samples.

## Supporting information

S1 File Supplementary methods used in “development and analytical validation of a novel bioavailable 25-hydroxyvitamin D assay”. (DOCX)

S1 Table DBP genotypes of study subjects. (DOCX)

S2 Table Digested DBP peptides used for quantification and genotyping of subjects and their tandem mass spectrometry mass transitions (parent and daughter ion m/z values). (DOCX)

S1 Fig Time course graphs comparing changes in concentrations of DBP, total 25OHD, and bioavailable 25OHD within patients 7–12 during the weeklong study period. (DOCX)

S2 Fig Time course graphs comparing changes in concentrations of DBP, total 25OHD, and bioavailable 25OHD within patients 13–18 during the weeklong study period. (DOCX)

S3 Fig Time course graphs comparing changes in concentrations of DBP, total 25OHD, and bioavailable 25OHD within patients 19–25 during the weeklong study period. (DOCX)

S4 Fig Comparison of DBP values measured by two LC-MS/MS assay methods for serum DBP after digestion with either Glu-C or trypsin protease on a separate cohort of patient samples. (DOCX)

S5 Fig Standard curve showing relationship between concentrations of DBP calibrator and concentrations of unbound (non-bioavailable) 25OHD tracer recovered from supernatant after separation from DBP-coated magnetic beads. (DOCX)

S6 Fig Relationship between % bioavailability of 25OHD in direct binding assay and concentrations of serum DBP and albumin in patient samples. (DOCX)

S7 Fig Relationship between % bioavailability of 25OHD in DBP-coated magnetic bead direct binding assay and % bioavailability in solid phase extraction direct binding assay (top panel); relationship between directly measured bioavailable 25OHD and calculated bioavailable 25OHD (bottom panel). (DOCX)

S8 Fig Relationship between % bioavailability of 25OHD in DBP-coated magnetic bead direct binding assay and directly measured 25OHD using a commercially available immunoassay. (DOCX)

## List of abbreviations

DBP: Vitamin D binding protein
25OHD_3_: 25-hydroxyvitamin D_3_
25OHD_2_: 25-hydroxyvitamin D_2_

## References

[1] Mendel CM. The free hormone hypothesis: a physiologically based mathematical model. Endocr Rev. 1989;10(3):232–74. doi: 10.1210/edrv-10-3-232 . 2673754

[2] Vermeulen A, Verdonck L, Kaufman JM. A critical evaluation of simple methods for the estimation of free testosterone in serum. J Clin Endocrinol Metab. 1999;84(10):3666–72. Epub 1999/10/16. doi: 10.1210/jcem.84.10.6079 . 10523012

[3] Bikle D, Bouillon R, Thadhani R, Schoenmakers I. Vitamin D metabolites in captivity? Should we measure free or total 25(OH)D to assess vitamin D status? J Steroid Biochem Mol Biol. 2017;173:105–16. doi: 10.1016/j.jsbmb.2017.01.007 . 28093353 PMC9005158

[4] Berg AH, Karumanchi SA, Thadhani R. Vitamin D-Binding Protein Deficiency and Homozygous Deletion of the GC Gene. N Engl J Med. 2019;380(26):2584–5. doi: 10.1056/NEJMc1905282 . 31242375 PMC6690847

[5] Faix JD. Principles and pitfalls of free hormone measurements. Best Pract Res Clin Endocrinol Metab. 2013;27(5):631–45. doi: 10.1016/j.beem.2013.06.007 . 24094635

[6] Daiger SP, Schanfield MS, Cavalli-Sforza LL. Group-specific component (Gc) proteins bind vitamin D and 25-hydroxyvitamin D. Proc Natl Acad Sci U S A. 1975;72(6):2076–80. doi: 10.1073/pnas.72.6.2076 ; PMCID: PMC432697. 49052 PMC432697

[7] Chun RF. New perspectives on the vitamin D binding protein. Cell Biochem Funct. 2012;30(6):445–56. doi: 10.1002/cbf.2835 . 22528806

[8] Henderson CM, Fink SL, Bassyouni H, Argiropoulos B, Brown L, Laha TJ, et al. Vitamin D-Binding Protein Deficiency and Homozygous Deletion of the GC Gene. N Engl J Med. 2019;380(12):1150–7. doi: 10.1056/NEJMoa1807841 . 30893535 PMC7898410

[9] Safadi FF, Thornton P, Magiera H, Hollis BW, Gentile M, Haddad JG, et al. Osteopathy and resistance to vitamin D toxicity in mice null for vitamin D binding protein. J Clin Invest. 1999;103(2):239–51. Epub 1999/01/23. doi: 10.1172/JCI5244 ; PMCID: 407885. 9916136 PMC407885

[10] Bikle DD, Schwartz J. Vitamin D Binding Protein, Total and Free Vitamin D Levels in Different Physiological and Pathophysiological Conditions. Front Endocrinol (Lausanne). 2019;10:317. doi: 10.3389/fendo.2019.00317 ; PMCID: PMC6546814. 31191450 PMC6546814

[11] Baer AN, Jan De Beur S. Vitamin D-Binding Protein Deficiency and Homozygous Deletion of the GC Gene. N Engl J Med. 2019;380(26):2582–3. doi: 10.1056/NEJMc1905282 . 31242372

[12] Bhan I, Powe CE, Berg AH, Ankers E, Wenger JB, Karumanchi SA, et al. Bioavailable vitamin D is more tightly linked to mineral metabolism than total vitamin D in incident hemodialysis patients. Kidney Int. 2012;82(1):84–9. doi: 10.1038/ki.2012.19 ; PMCID: PMC3376220. 22398410 PMC3376220

[13] Powe CE, Ricciardi C, Berg AH, Erdenesanaa D, Collerone G, Ankers E, et al. Vitamin D-binding protein modifies the vitamin D-bone mineral density relationship. J Bone Miner Res. 2011;26(7):1609–16. Epub 2011/03/19. doi: 10.1002/jbmr.387 ; PMCID: 3351032. 21416506 PMC3351032

[14] Yao P, Sun L, Lu L, Ding H, Chen X, Tang L, et al. Effects of Genetic and Nongenetic Factors on Total and Bioavailable 25(OH)D Responses to Vitamin D Supplementation. J Clin Endocrinol Metab. 2017;102(1):100–10. doi: 10.1210/jc.2016-2930 . 27768857

[15] Botella-Carretero JI, Lafuente C, Montes-Nieto R, Balsa J, Vega-Pinero B, Garcia-Moreno F, et al. Serum Bioavailable Vitamin D Concentrations and Bone Mineral Density in Women After Obesity Surgery. Obes Surg. 2016;26(11):2732–7. doi: 10.1007/s11695-016-2185-1 . 27116295

[16] Aggarwal A, Yadav AK, Ramachandran R, Kumar V, Kumar V, Sachdeva N, et al. Bioavailable vitamin D levels are reduced and correlate with bone mineral density and markers of mineral metabolism in adults with nephrotic syndrome. Nephrology (Carlton). 2016;21(6):483–9. doi: 10.1111/nep.12638 . 26428957

[17] Yu C, Xue H, Wang L, Chen Q, Chen X, Zhang Y, et al. Serum Bioavailable and Free 25-Hydroxyvitamin D Levels, but Not Its Total Level, Are Associated With the Risk of Mortality in Patients With Coronary Artery Disease. Circ Res. 2018;123(8):996–1007. doi: 10.1161/CIRCRESAHA.118.313558 . 30355032

[18] Powe CE, Evans MK, Wenger J, Zonderman AB, Berg AH, Nalls M, et al. Vitamin D-binding protein and vitamin D status of black Americans and white Americans. N Engl J Med. 2013;369(21):1991–2000. Epub 2013/11/22. doi: 10.1056/NEJMoa1306357 ; PMCID: 4030388. 24256378 PMC4030388

[19] Chen Y. Vitamin D-binding protein and vitamin D in blacks and whites. N Engl J Med. 2014;370(9):878–9. Epub 2014/02/28. doi: 10.1056/NEJMc1315850 . 24571764

[20] Bouillon R, Jones K, Schoenmakers I. Vitamin D-binding protein and vitamin D in blacks and whites. N Engl J Med. 2014;370(9):879. Epub 2014/02/28. doi: 10.1056/NEJMc1315850 . 24571765

[21] Braun A, Brandhofer A, Cleve H. Interaction of the vitamin D-binding protein (group-specific component) and its ligand 25-hydroxy-vitamin D3: binding differences of the various genetic types disclosed by isoelectric focusing. Electrophoresis. 1990;11(6):478–83. doi: 10.1002/elps.1150110608 . 2394211

[22] Arnaud J, Constans J. Affinity differences for vitamin D metabolites associated with the genetic isoforms of the human serum carrier protein (DBP). Hum Genet. 1993;92(2):183–8. Epub 1993/09/01. doi: 10.1007/BF00219689 . 8370586

[23] Boutin B, Galbraith RM, Arnaud P. Comparative affinity of the major genetic variants of human group-specific component (vitamin D-binding protein) for 25-(OH) vitamin D. J Steroid Biochem. 1989;32(1A):59–63. doi: 10.1016/0022-4731(89)90014-9 . 2913402

[24] Bikle DD, Gee E, Halloran B, Kowalski MA, Ryzen E, Haddad JG. Assessment of the free fraction of 25-hydroxyvitamin D in serum and its regulation by albumin and the vitamin D-binding protein. J Clin Endocrinol Metab. 1986;63(4):954–9. Epub 1986/10/01. doi: 10.1210/jcem-63-4-954 . 3745408

[25] Denburg MR, Hoofnagle AN, Sayed S, Gupta J, de Boer IH, Appel LJ, et al. Chronic Renal Insufficiency Cohort study i. Comparison of Two ELISA Methods and Mass Spectrometry for Measurement of Vitamin D-Binding Protein: Implications for the Assessment of Bioavailable Vitamin D Concentrations Across Genotypes. J Bone Miner Res. 2016;31(6):1128–36. doi: 10.1002/jbmr.2829 ; PMCID: PMC4945118. 27250744 PMC4945118

[26] Jemielita TO, Leonard MB, Baker J, Sayed S, Zemel BS, Shults J, et al. Association of 25-hydroxyvitamin D with areal and volumetric measures of bone mineral density and parathyroid hormone: impact of vitamin D-binding protein and its assays. Osteoporos Int. 2016;27(2):617–26. doi: 10.1007/s00198-015-3296-6 ; PMCID: PMC4924926. 26359185 PMC4924926

[27] Wang YS, Hershman JM, Pekary AE. Improved ultrafiltration method for simultaneous measurement of free thyroxin and free triiodothyronine in serum. Clin Chem. 1985;31(4):517–22. . 3978781

[28] van Hoof HJ, Swinkels LM, Ross HA, Sweep CG, Benraad TJ. Determination of non-protein-bound plasma 1,25-dihydroxyvitamin D by symmetric (rate) dialysis. Anal Biochem. 1998;258(2):176–83. doi: 10.1006/abio.1998.2586 . 9570827

[29] Pettifor JM, Bikle DD, Cavaleros M, Zachen D, Kamdar MC, Ross FP. Serum levels of free 1,25-dihydroxyvitamin D in vitamin D toxicity. Ann Intern Med. 1995;122(7):511–3. doi: 10.7326/0003-4819-122-7-199504010-00006 . 7872586

[30] Berg AH, Powe CE, Evans MK, Wenger J, Ortiz G, Zonderman AB, et al. 24,25-Dihydroxyvitamin d3 and vitamin D status of community-dwelling black and white Americans. Clin Chem. 2015;61(6):877–84. doi: 10.1373/clinchem.2015.240051 ; PMCID: PMC4686255. 25922442 PMC4686255

[31] Heureux N, Lindhout E, Swinkels L. A Direct Assay for Measuring Free 25-Hydroxyvitamin D. J AOAC Int. 2017;100(5):1318–22. doi: 10.5740/jaoacint.17-0084 . 28492143

[32] Smith LM, Gallagher JC. Effect of vitamin D supplementation on total and free 25 hydroxyvitamin D and parathyroid hormone. An analysis of two randomized controlled trials. J Intern Med. 2019. doi: 10.1111/joim.12950 . 31215092 PMC6851404

[33] Meier U, Gressner O, Lammert F, Gressner AM. Gc-globulin: roles in response to injury. Clin Chem. 2006;52(7):1247–53. doi: 10.1373/clinchem.2005.065680 . 16709624

[34] Binkley N, Coursin D, Krueger D, Iglar P, Heiner J, Illgen R, et al. Surgery alters parameters of vitamin D status and other laboratory results. Osteoporos Int. 2017;28(3):1013–20. doi: 10.1007/s00198-016-3819-9 . 27826645

[35] Dahl B, Schiodt FV, Gehrchen PM, Ramlau J, Kiaer T, Ott P. Gc-globulin is an acute phase reactant and an indicator of muscle injury after spinal surgery. Inflamm Res. 2001;50(1):39–43. doi: 10.1007/s000110050722 . 11235020

[36] Czarnik T, Czarnik A, Gawda R, Gawor M, Piwoda M, Marszalski M, et al. Vitamin D kinetics in the acute phase of critical illness: A prospective observational study. J Crit Care. 2018;43:294–9. doi: 10.1016/j.jcrc.2017.09.179 . 28968524

[37] Amrein K, Christopher KB, McNally JD. Understanding vitamin D deficiency in intensive care patients. Intensive Care Med. 2015;41(11):1961–4. doi: 10.1007/s00134-015-3937-4 . 26142054

[38] Amrein K, Schnedl C, Holl A, Riedl R, Christopher KB, Pachler C, et al. Effect of high-dose vitamin D3 on hospital length of stay in critically ill patients with vitamin D deficiency: the VITdAL-ICU randomized clinical trial. JAMA. 2014;312(15):1520–30. doi: 10.1001/jama.2014.13204 . 25268295

[39] Best CM, Pressman EK, Queenan RA, Cooper E, O’Brien KO. Longitudinal changes in serum vitamin D binding protein and free 25-hydroxyvitamin D in a multiracial cohort of pregnant adolescents. J Steroid Biochem Mol Biol. 2019;186:79–88. doi: 10.1016/j.jsbmb.2018.09.019 ; PMCID: PMC6611677. 30278215 PMC6611677

[40] Schwartz JB, Gallagher JC, Jorde R, Berg V, Walsh J, Eastell R, et al. Determination of Free 25(OH)D Concentrations and Their Relationships to Total 25(OH)D in Multiple Clinical Populations. J Clin Endocrinol Metab. 2018;103(9):3278–88. doi: 10.1210/jc.2018-00295 ; PMCID: PMC6126881. 29955795 PMC6126881

[41] Lutsey PL, Parrinello CM, Misialek JR, Hoofnagle AN, Henderson CM, Laha TJ, et al. Short-term Variability of Vitamin D-Related Biomarkers. Clin Chem. 2016;62(12):1647–53. doi: 10.1373/clinchem.2016.261461 ; PMCID: PMC5131784. 27694392 PMC5131784

