# Supplementary material for: Development and analytical validation of a novel bioavailable 25-hydroxyvitamin D assay
Source: PLoS One. 2021 Jul 9;16(7):e0254158. doi: 10.1371/journal.pone.0254158 (PMC8270209; doi:10.1371/journal.pone.0254158)
Supplement: S1 File — (DOCX) [file pone.0254158.s001.docx]

Supporting Information for “Development and Analytical Validation of a Novel Bioavailable 25-hydroxyvitamin D Assay”

Anders H. Berg, MD, PhD^1*^; Mahtab Tavasoli, PhD^2^; Agnes S. Lo, PhD^3,4^, Sherri-Ann M. Burnett-Bowie, MD, MPH^5^, Ishir Bhan, MD, MPH^6^, S. Ananth Karumanchi, MD^3,4^; Sahir Kalim, MD, MPH^6^; Dongsheng Zhang, PhD^3,4^, Sophia Zhao, MD^6^; Ravi I. Thadhani, MD, MPH^6^

^1^Department of Pathology, Cedars Sinai Medical Center, Los Angeles, California, United States of America

^2^Department of Pathology, Beth Israel Deaconess Medical Center and Harvard Medical School, Boston, Massachusetts, United States of America

^3^Departments of Medicine and Biomedical Sciences, Cedars-Sinai Medical Center, Los Angeles, California, United States of America

^4^Division of Nephrology and Center for Vascular Biology Research, Department of Medicine, Beth Israel Deaconess Medical Center and Harvard Medical School, Boston, Massachusetts, United States of America

^5^Endocrine Division, Department of Medicine, Massachusetts General Hospital, Boston, Massachusetts, United States of America

^6^Division of Nephrology, Department of Medicine, Massachusetts General Hospital, Boston, Massachusetts, United States of America

*Corresponding Author

Email: [anders.berg@cshs.org](mailto:anders.berg@cshs.org)

**SUPPLEMENTARY METHODS**

**Measurement of serum Total 25-hydroxyvitamin D3 and 25-hydroxyvitamin D2**

Blood samples were drawn into serum separator tubes without anticoagulant, centrifuged at 1430 x g for 15 minutes, and stored at -80ºC for future analysis. Concentrations of total 25OHD_3_ and 25OHD_2_ were measured from 100 µL of serum using LC-MS/MS using previously published methods and reagents. [43] For these analyses, was mixed with 25OHD_3_–[^2^H_6_] isotopic internal standards dissolved in 5% bovine serum albumin (IsoSciences, Inc., King of Prussia, PA) and 25OHD_3,_ 25OHD_2_, were isolated by solid phase extraction (Strata C-18E 96-well SPE plates, Phenomenex, Inc., Torrence, CA), eluted with acetonitrile and derivatized with 4-phenyl-1,2,4-triazole-3,5-dione (PTAD). Samples were vacuum lyophilized and redissolved with 100 µL of 50% ethanol. Samples were then analyzed for vitamin D metabolites using reverse phase chromatography coupled to tandem mass spectrometry in multiple reaction monitoring mode (intra-assay CV: 1.1%, 1.3% for 25OHD_3_, 25OHD_2_, respectively). Assays were calibrated using 25OHD3 and 25OHD2 Certified Reference Standards (Cerilliant, Inc., Round Rock, TX; accuracy traceable to NIST SRM 2972 reference material). Complete descriptions of chromatography and mass spectrometer settings for total 25OHD measurements have been previously described.

**Measurement of serum DBP**

Serum DBP concentrations and genotyping were measured using proteolytic digestions followed by LC-MS/MS by methods previously described. [44] Briefly, 10 microliters patient serum were mixed with 10 microliters pooled bovine serum in order to use bovine DBP as an internal standard to ensure 100% digestion efficiency and analytical yield. 2 microliters of sample and internal standard were then diluted into 100 microliters digestion mixture (0.1 mg/mL Glu-C endoproteinase, 50 mM ammonium bicarbonate, 10 mM dithiothreitol) and digested overnight at room temperature. 10 microliters of this digestion were injected for LC-MS/MS analysis and analyzed for the quantification and variant-specific genotyping peptides shown in S1 Table. The DBP genotypes of the subjects are shown in S2 Table. The assay was validated by comparison to parallel measurements using the same samples digested with trypsin and analyzed by LC-MS/MS using previously published methods.[45] The Glu-C assay is our preferred assay method because of ease of digestion. There was 100% concordance between the genotypes identified using the two methods. The LC-MS/MS quantitative assay for DBP using Glu-C digestion was in excellent agreement with the trypsin assay method (S4 Fig, r = +0.9116, p<0.0001).

**Development and validation of direct assay for Bioavailable 25OHD**

Previous studies of bioavailable 25OHD have estimated concentrations by measuring serum DBP, albumin, and 25OHD concentrations and calculating bioavailable 25OHD concentrations based upon published estimates of the affinity binding constants between 25OHD and DBP. Evidence suggests that the affinity of DBP for 25OHD differs between the three different DBP protein genetic variants (Gc1S, Gc1F, Gc2), however.[46] Furthermore, previous studies estimating the affinity binding constants for the different protein variants have produced differing affinities, making the true affinity binding characteristics difficult to define with confidence. [46-49] Lastly, since we cannot know how much of the total of each variant is expressed in heterozygous patients (e.g. 70% Gc1S and 30% Gc1F), it is not possible to accurately calculate concentrations of bioavailable 25OHD in patients who are heterozygous and express two different variants. For these reasons, there is consensus that estimating concentrations of free and/or bioavailable 25OHD based on these calculated methods is suboptimal, and there is need for direct methods for measurement of free and/or bioavailable 25OHD which respond to differences in the concentration and binding affinities of DBP (as well as other serum 25OHD binding factors). [50]

Towards this end, we developed a novel direct assay that measures the proportion (%) of bioavailable 25OHD using the principle of affinity chromatography. A fixed amount of 25OHD_3_ stable isotopic tracer is mixed with dilute serum, and the % bioavailable 25OHD is defined as the fraction of 25OHD isotopic tracer that binds to the DBP-coated magnetic beads under physiologic binding conditions. The assay is calibrated using standards containing increasing concentrations of purified DBP (Sigma Aldrich, St. Louis, MO). Binding reactions were mixed in 96-well microtiters plates, left 10 minutes at room temperature to allow for binding equilibrium, then beads are magnetically separated and the supernatant containing non-bioavailable 25OHD tracer is aspirated off the beads and tracer is extracted by solid phase extraction on a Strata-X 96-well 2 mg microelution plate (Phenomenex Inc., Torrance, CA). Extracted 25OHD tracer was then measured by LC-MS/MS on an API 5000 triple quadrupole mass spectrometer (AB Sciex LLC, Framingham, MA) using previously described methods [43]. DBP-coated magnetic beads (Dynabeads M-270 Epoxy functionalized) were made according to the manufacturer’s instructions by binding purified DBP (Human Gc globin from Sigma Aldrich, St. Louis, MO) at a concentration of 30 micrograms per millgram of beads. After covalent linkage, beads were inactivated with 50 mM Tris-HCl pH 7.4, washed with PBS and 0.1% Tween 20 three times to remove unbound DBP, then resuspended in Tris-buffered saline pH 7.4 at 10 mg/mL for use. Each 96-well plate of reactions was calibrated using 6 calibrators containing 5% bovine serum albumin and 0, 100, 200, 300, 400, 500 micrograms/mL purified DBP purified human DBP (Human Gc globin from Sigma Aldrich, St. Louis, MO) diluted in Tris-buffered saline pH 7.4, aliquoted and frozen at -80°C for future use.

As seen in S5 Fig, the relationship between the amount of non-bead bound non-bioavailable 25OHD tracer recovered from the supernatant and the concentration of DBP present in the calibrator is linear. Because this competitive assay responds to all factors present in the patient’s serum that bind 25OHD, the proportion of 25OHD bound to the beads responds to differences in the concentration of DBP and differences in the affinity of DBP variants present, and also accounts for binding to albumin, lipoproteins, or any other high affinity binding factors that may be present. In order the validate the responsiveness of the assay to differences in DBP concentrations in patient samples, % bioavailable 25OHD measurements were plotted against serum concentrations of DBP. As shown in S6 Fig, directly measured % bioavailable 25OHD values were inversely correlated with concentrations of DBP (r = -0.7130, p<0.001), but were not significantly correlated to serum albumin concentrations. Using this direct assay method, we are then able to calculate absolute concentrations of bioavailable 25OHD from measured [Total 25OHD] × %Bioavailable 25OHD.

In order to validate our direct assay for bioavailable 25OHD, measurements were compared to calculated bioavailable 25OHD concentrations, compared to an alternate direct assay for bioavailable 25OHD, and compared to directly measured free 25OHD concentrations. As shown in S7 Fig, % bioavailable 25OHD values were directly proportional to values obtained using a previously published solid phase extraction assay method (r = +0.9159, p<0.0001) [44], and absolute concentrations of directly measured bioavailable 25OHD were in close agreement with calculated bioavailable 25OHD concentration (r = +0.9339, p<0.0001). Concentrations of bioavailable 25OHD were also significantly correlated to concentrations of free 25OHD measured by a commercial immunoassay method developed by Future Diagnostics, LLC (S8 Fig, r = +0.5765, p<0.001).

References

43. Berg, A.H., et al., *24,25-Dihydroxyvitamin d3 and vitamin D status of community-dwelling black and white Americans.* Clin Chem, 2015. **61**(6): p. 877-84.

44. Berg, A.H., et al., *Acute Homeostatic Changes Following Vitamin D2 Supplementation.* J Endocr Soc, 2017. **1**(9): p. 1135-1149.

45. Hoofnagle, A.N., J.H. Eckfeldt, and P.L. Lutsey, *Vitamin D-Binding Protein Concentrations Quantified by Mass Spectrometry.* N Engl J Med, 2015. **373**(15): p. 1480-2.

46. Arnaud, J. and J. Constans, *Affinity differences for vitamin D metabolites associated with the genetic isoforms of the human serum carrier protein (DBP).* Hum Genet, 1993. **92**(2): p. 183-8.

47. Bikle, D.D., et al., *Assessment of the free fraction of 25-hydroxyvitamin D in serum and its regulation by albumin and the vitamin D-binding protein.* J Clin Endocrinol Metab, 1986. **63**(4): p. 954-9.

48. Bouillon, R., H. van Baelen, and P. de Moor, *Comparative study of the affinity of the serum vitamin D-binding protein.* J Steroid Biochem, 1980. **13**(9): p. 1029-34.

49. Chun, R.F., et al., *Vitamin D binding protein and monocyte response to 25-hydroxyvitamin D and 1,25-dihydroxyvitamin D: analysis by mathematical modeling.* PLoS One, 2012. **7**(1): p. e30773.

50. Bikle, D.D., S. Malmstroem, and J. Schwartz, *Current Controversies: Are Free Vitamin Metabolite Levels a More Accurate Assessment of Vitamin D Status than Total Levels?* Endocrinol Metab Clin North Am, 2017. **46**(4): p. 901-918.
