# Supplementary material for: Development and analytical validation of a novel bioavailable 25-hydroxyvitamin D assay
Source: PLoS One. 2021 Jul 9;16(7):e0254158. doi: 10.1371/journal.pone.0254158 (PMC8270209; doi:10.1371/journal.pone.0254158)
Supplement: S1 Table — (DOCX) [file pone.0254158.s002.docx]

| **Genotype** | **No. of subjects** |
| --- | --- |
| Gc1S/Gc1S | 9 |
| Gc1S/Gc1F | 8 |
| Gc1S/Gc2 | 7 |
| Gc1F/Gc2 | 1 |
| Gc1F/Gc1F | 0 |
| Gc2/Gc2 | 0 |
