# Supplementary material for: Development and analytical validation of a novel bioavailable 25-hydroxyvitamin D assay
Source: PLoS One. 2021 Jul 9;16(7):e0254158. doi: 10.1371/journal.pone.0254158 (PMC8270209; doi:10.1371/journal.pone.0254158)
Supplement: S2 Table — (DOCX) [file pone.0254158.s003.docx]

| Peptide ID | Peptide Sequence | Parent ion  (m/z) | Daughter ion  (m/z) |
| --- | --- | --- | --- |
| Human DBP Quantitation | SNSPFPVHPGTAE | 670.3 | 807.4 |
| Human DBP quant confirmation | LSRRTHLPE | 554.8 | 864.5 |
| Bovine DBP internal standard | SNSPFPVHPGTPE | 683.3 | 833.4 |
| Gc1S variant | RLKAKLPE | 478.1 | 597.4 |
| Gc1F variant | RLKAKLPDATPTE | 480.6 | 710.5 |
| Gc2 variant | RLKAKLPDATPKE | 489.9 | 597.4 |
