# Supplementary figures and images for: Development and analytical validation of a novel bioavailable 25-hydroxyvitamin D assay
Source: PLoS One. 2021 Jul 9;16(7):e0254158. doi: 10.1371/journal.pone.0254158 (PMC8270209; doi:10.1371/journal.pone.0254158)

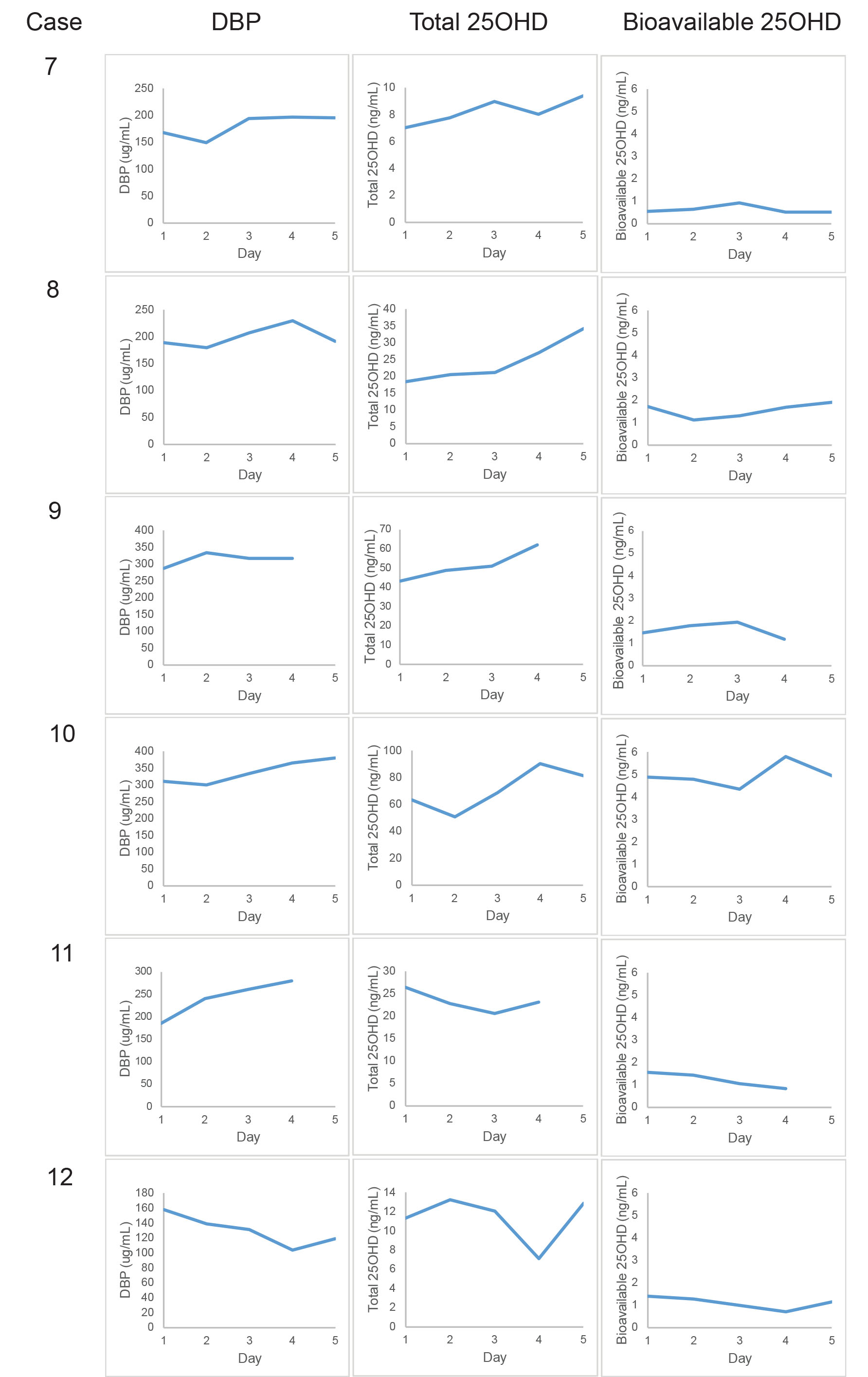

Supplement: S1 Fig — (DOCX) [file pone.0254158.s004.docx]

**
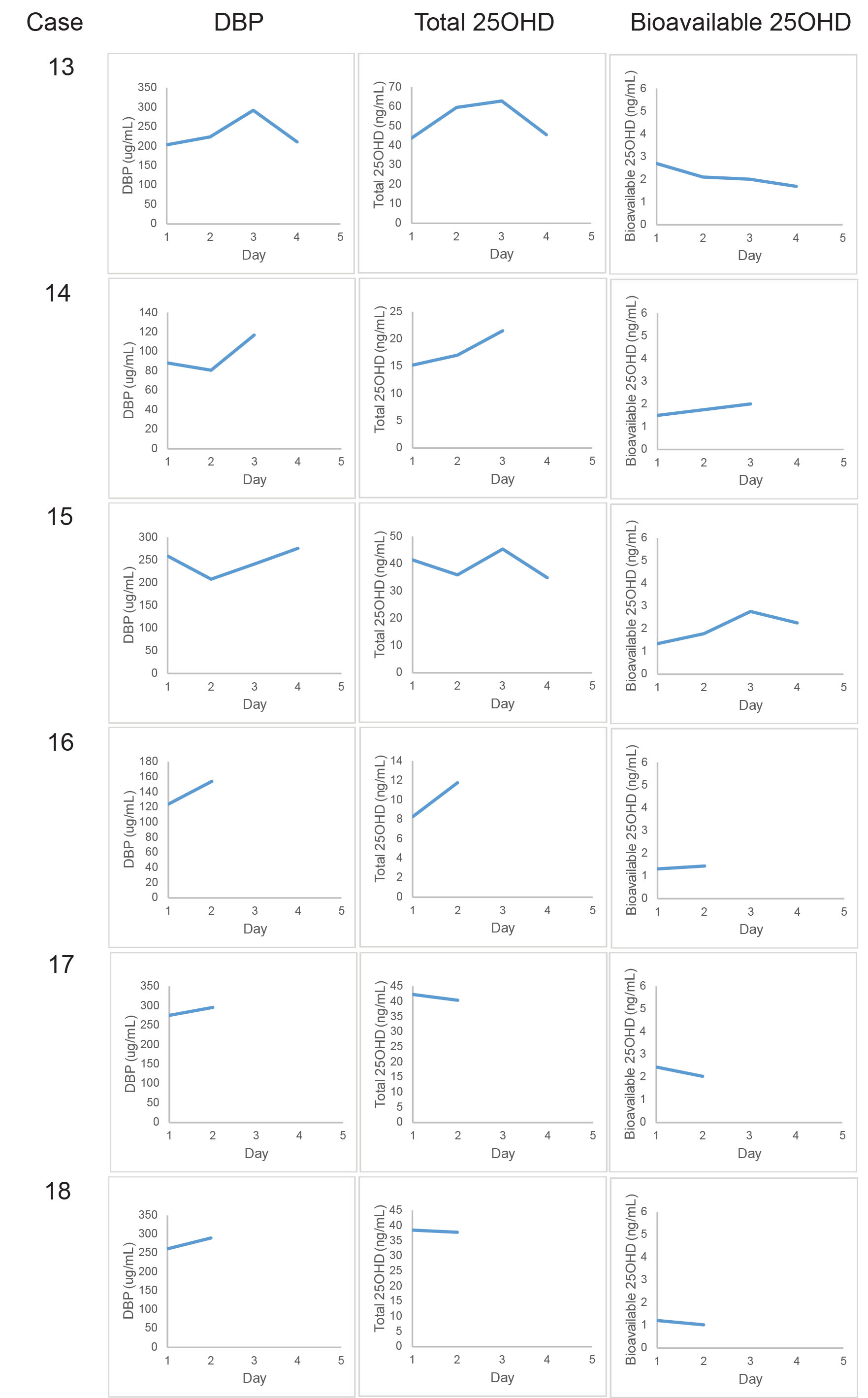
**

Supplement: S2 Fig — (DOCX) [file pone.0254158.s005.docx]

**
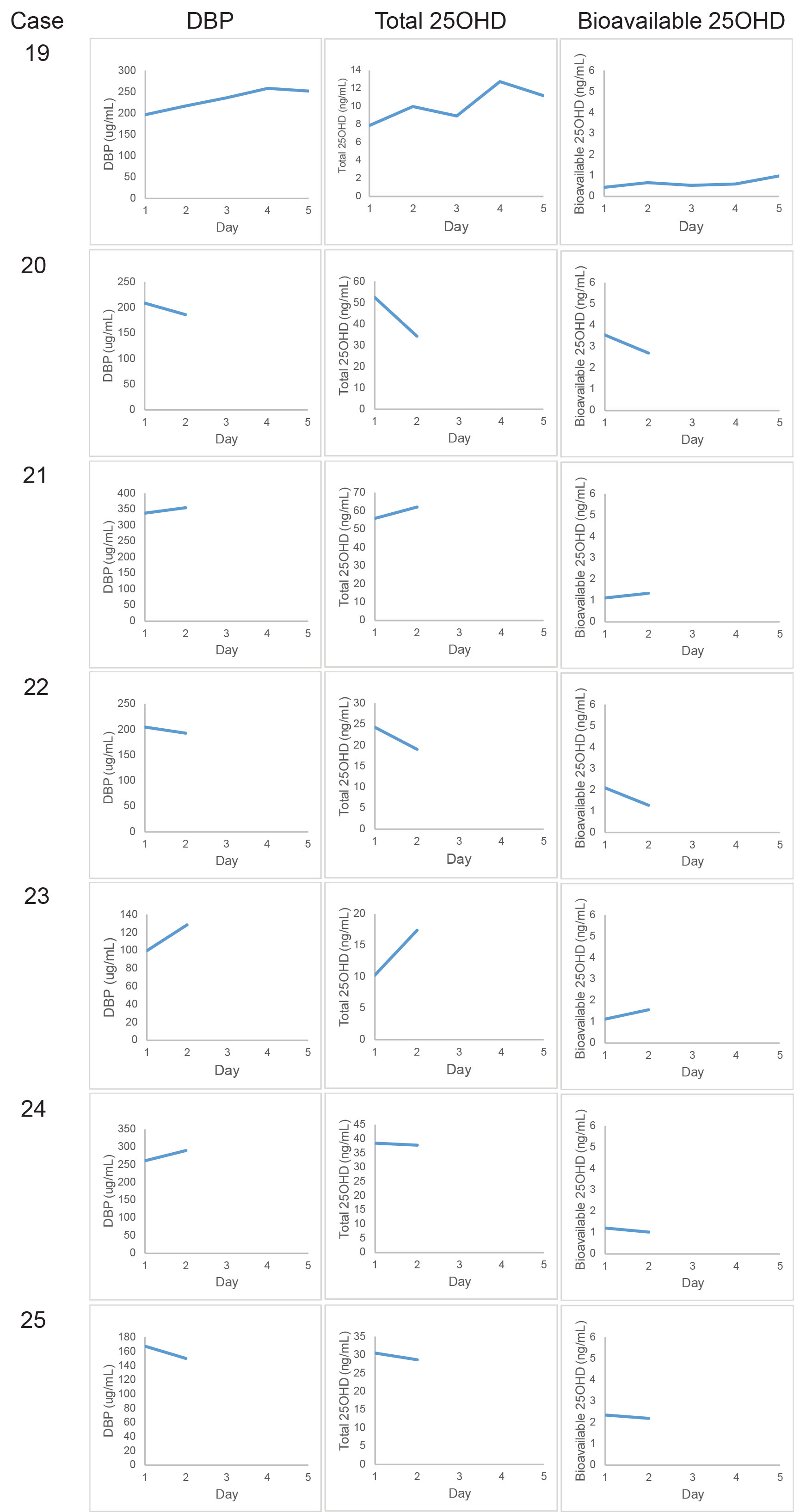
**

Supplement: S3 Fig — (DOCX) [file pone.0254158.s006.docx]

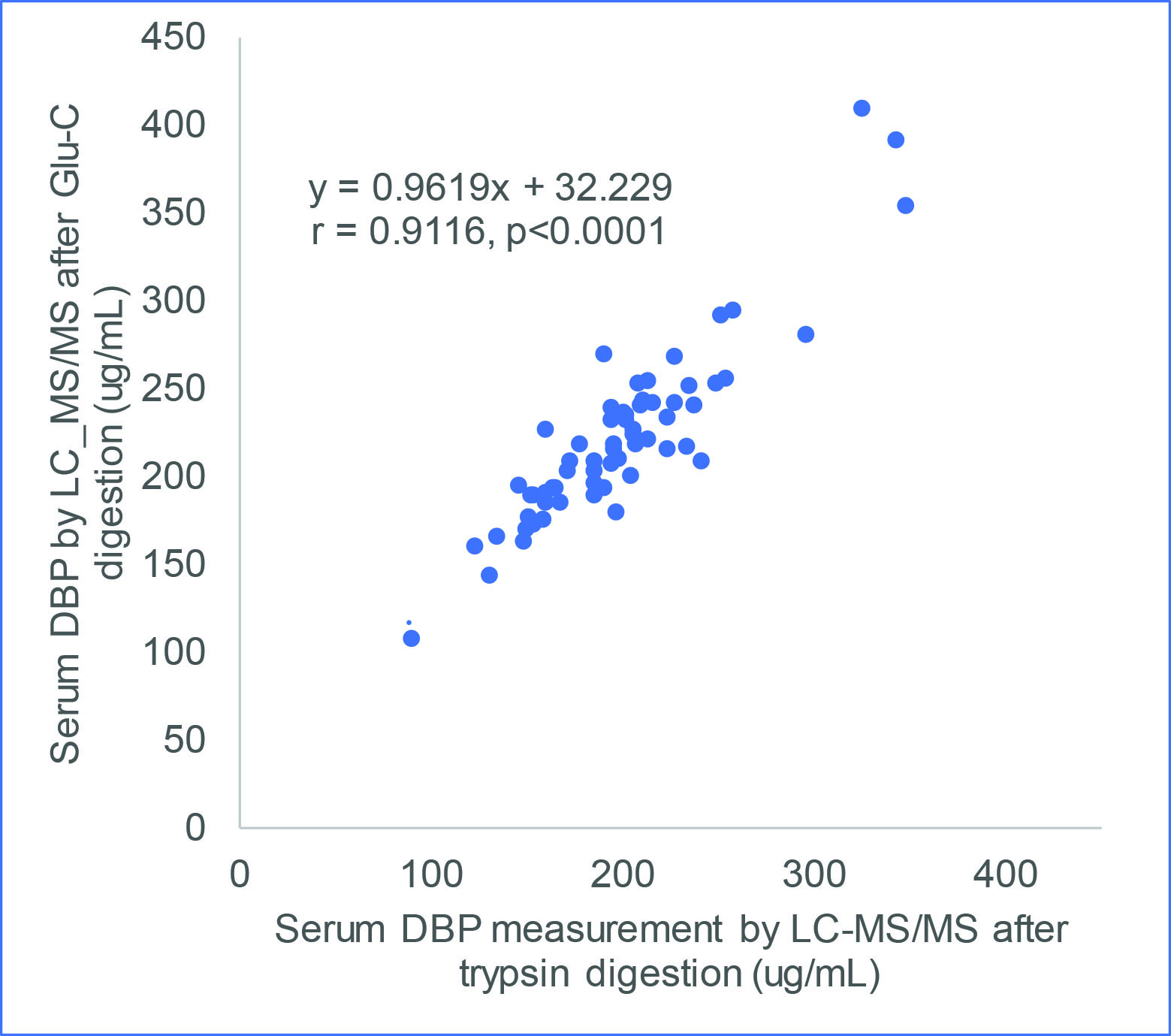

Supplement: S4 Fig — (DOCX) [file pone.0254158.s007.docx]

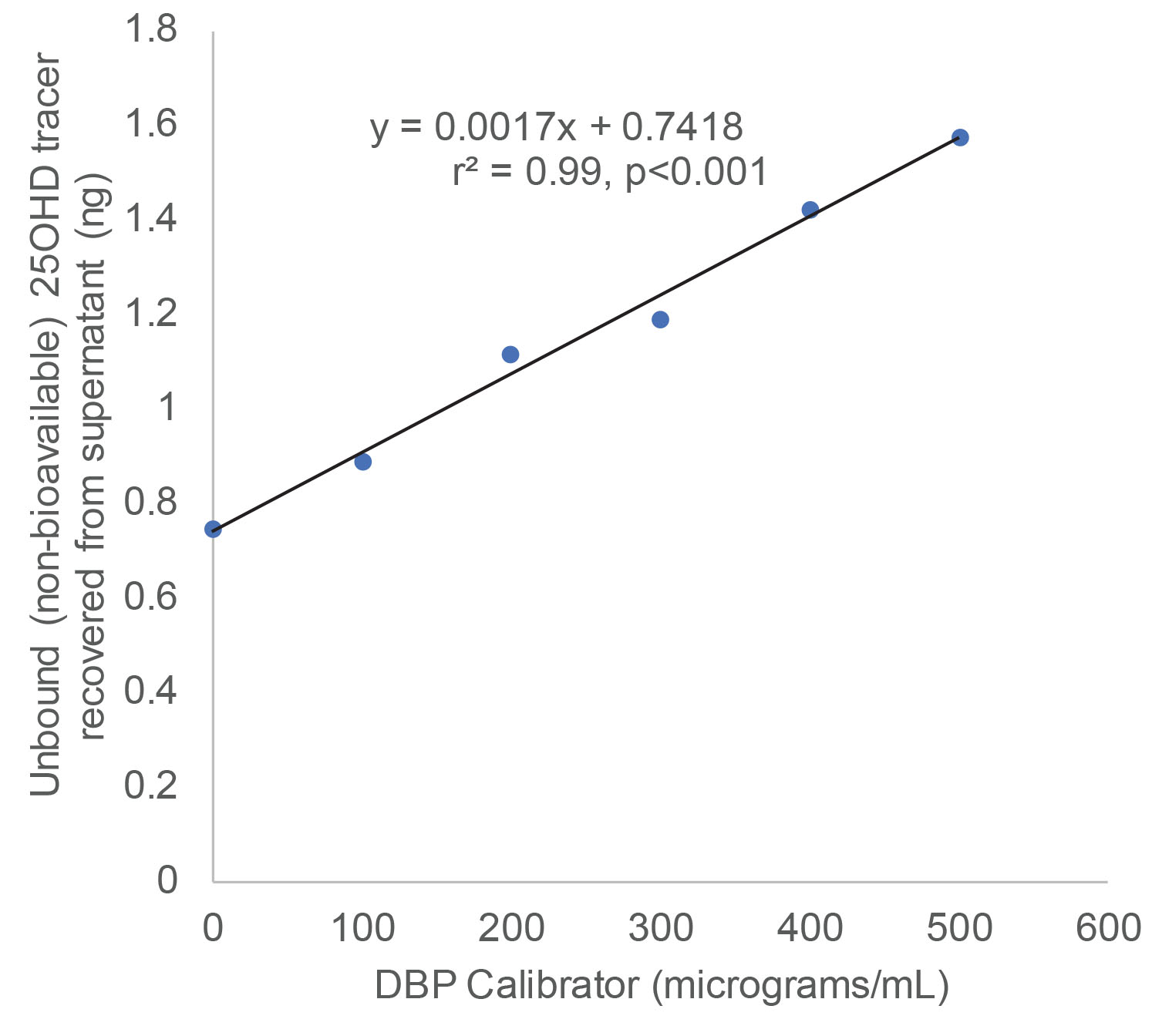

Supplement: S5 Fig — (DOCX) [file pone.0254158.s008.docx]

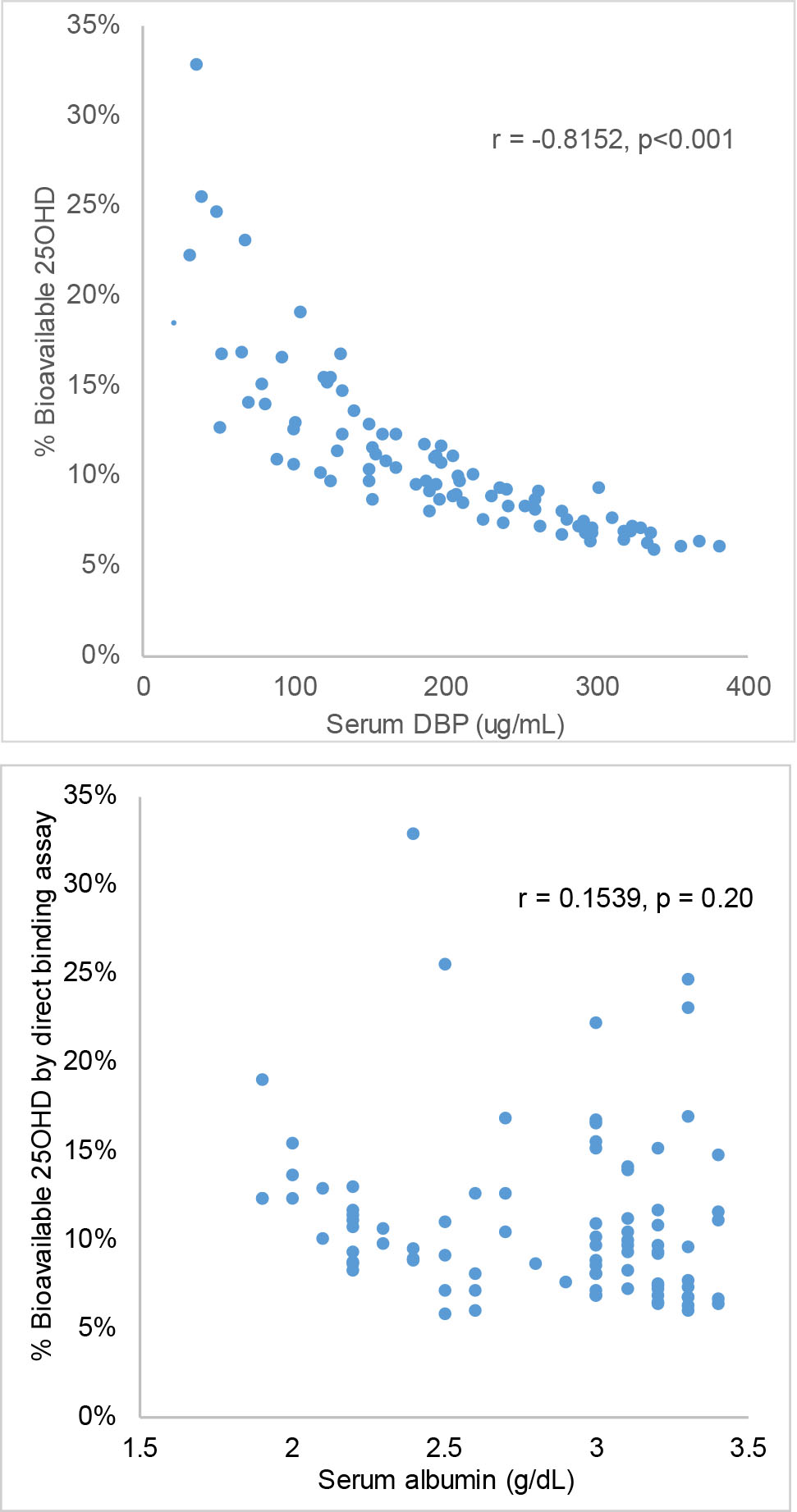

Supplement: S6 Fig — (DOCX) [file pone.0254158.s009.docx]

**
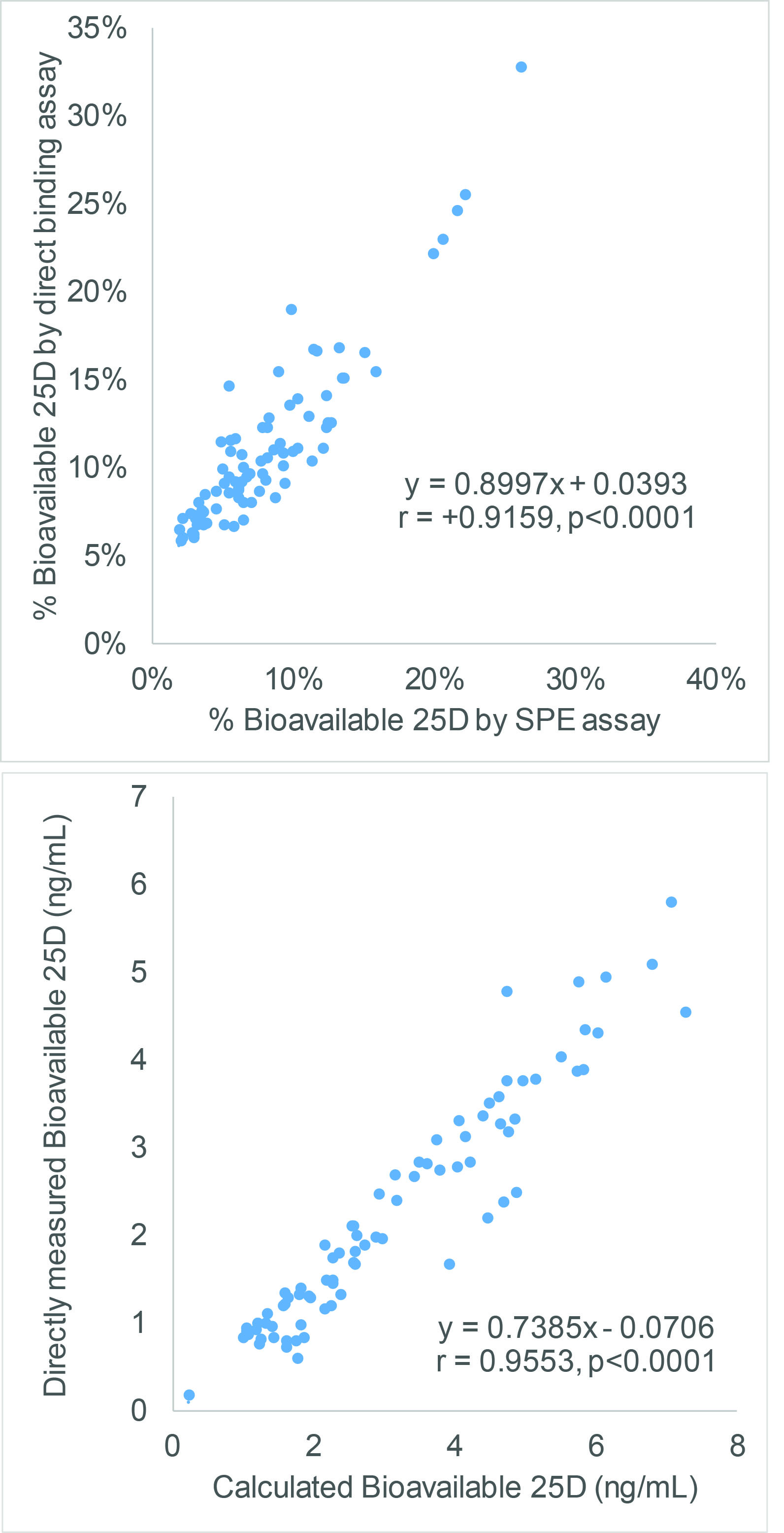
**

Supplement: S7 Fig — (DOCX) [file pone.0254158.s010.docx]

**
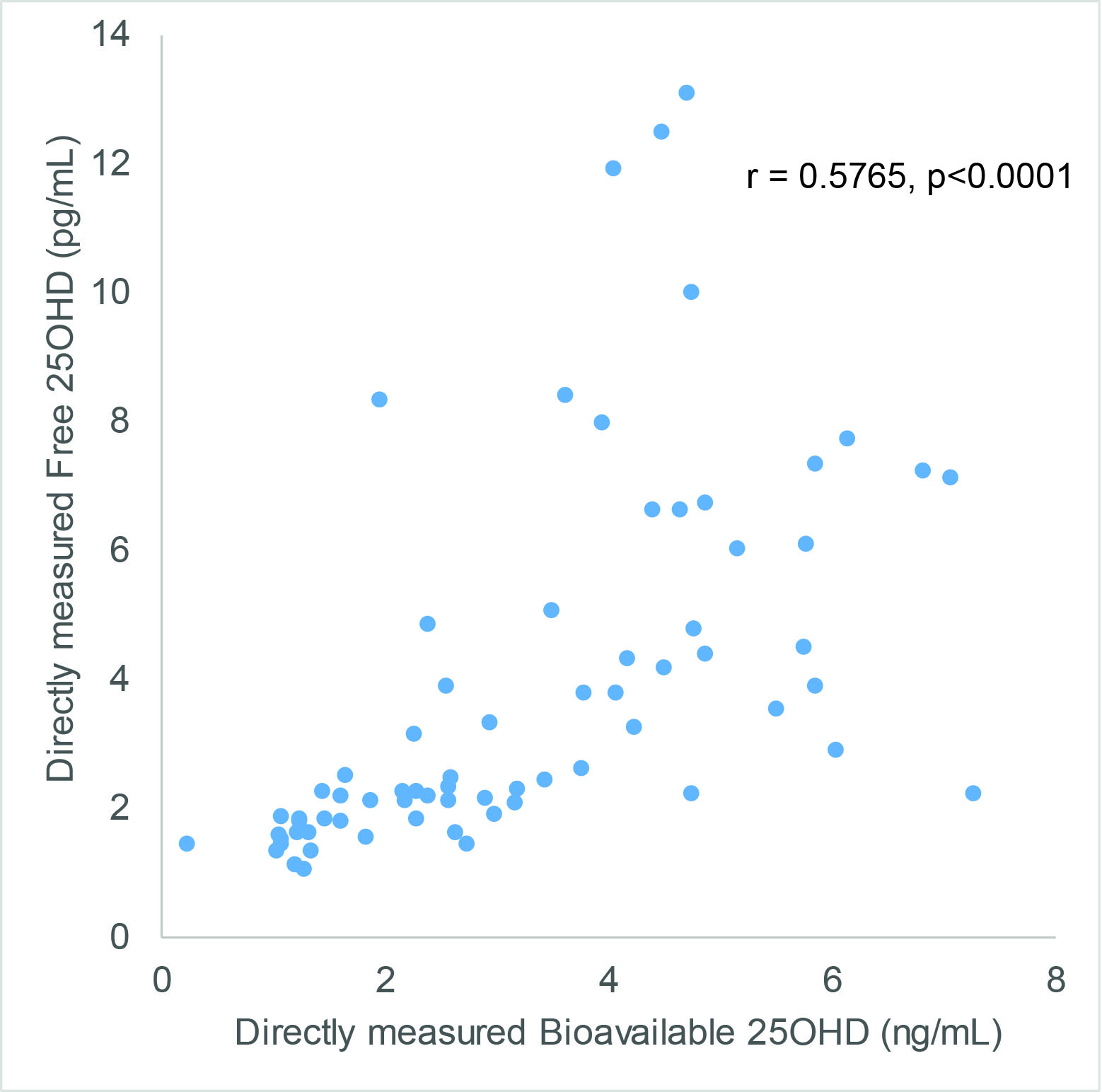
**

Supplement: S8 Fig — (DOCX) [file pone.0254158.s011.docx]
